# HetIG-PreDiG: A Heterogeneous Integrated Graph Model for Predicting Human Disease Genes based on gene expression

**DOI:** 10.1371/journal.pone.0280839

**Published:** 2023-02-15

**Authors:** Kathleen M. Jagodnik, Yael Shvili, Alon Bartal

**Affiliations:** 1 The School of Business Administration, Bar-Ilan University, Ramat Gan, Israel; 2 Department of Psychiatry, Harvard Medical School, Boston, MA, United States of America; 3 Department of Psychiatry, Massachusetts General Hospital, Boston, MA, United States of America; 4 Department of Surgery A, Meir Medical Center, Kfar Sava, Israel; Koc Universitesi, TURKEY

## Abstract

Graph analytical approaches permit identifying novel genes involved in complex diseases, but are limited by (i) inferring structural network similarity of connected gene nodes, ignoring potentially relevant unconnected nodes; (ii) using homogeneous graphs, missing gene-disease associations’ complexity; (iii) relying on disease/gene-phenotype associations’ similarities, involving highly incomplete data; (iv) using binary classification, with gene-disease edges as positive training samples, and non-associated gene and disease nodes as negative samples that may include currently unknown disease genes; or (v) reporting predicted novel associations without systematically evaluating their accuracy. Addressing these limitations, we develop the Heterogeneous Integrated Graph for Predicting Disease Genes (HetIG-PreDiG) model that includes gene-gene, gene-disease, and gene-tissue associations. We predict novel disease genes using low-dimensional representation of nodes accounting for network structure, and extending beyond network structure using the developed Gene-Disease Prioritization Score (GDPS) reflecting the degree of gene-disease association via gene co-expression data. For negative training samples, we select non-associated gene and disease nodes with lower GDPS that are less likely to be affiliated. We evaluate the developed model’s success in predicting novel disease genes by analyzing the prediction probabilities of gene-disease associations. HetIG-PreDiG successfully predicts (Micro-F1 = 0.95) gene-disease associations, outperforming baseline models, and is validated using published literature, thus advancing our understanding of complex genetic diseases.

## 1 Introduction

Understanding the complex biological phenomena involved in human diseases is essential for developing new preventive and therapeutic strategies [1]. Since authoritative sets of genetic associations for many diseases are unknown [1], and the experimentation necessary to validate these associations is costly and time consuming, researchers have developed computational methods, including machine learning (ML) models to discover gene-disease associations [2]. Analyzing biological data using graphs can identify complex interactions among entities (e.g., genes and diseases), and it facilitates the detection of variations (e.g., genetic mutations) via structural changes in the graph [3–5].

Some disease gene prediction models [6] assume that genes associated with biologically similar diseases have similar graph structures. Those models miss potentially relevant nodes beyond the local neighborhood of a node. Network diffusion models extend beyond the local neighborhood of known disease genes by walking over the edges of a biological graph [7–11]. For example, the MEXCOwalk algorithm [11] performs an edge-weighted random walk on a graph to identify cancer gene modules, and the HotNet2 algorithm [8, 9] employs a directed network diffusion model to assess the significance of mutations in genes, and the local topology of interactions among encoded proteins, to identify mutated subnetworks in a genome-scale interaction network. However, network diffusion models that apply walks over the graph miss potentially relevant nodes that are unconnected (e.g., isolated components or nodes) due to, e.g., missing or unknown data. For example, Del Sol et al. [12] reported that complete miRNA networks accurately represent healthy tissues, whereas cancer tissues are characterized by disjointed, disconnected sub-networks. Additionally, the lack of data about gene–gene associations is often the source of the “missing heritability” problem in which known interactions can explain only a small portion of a disease [13]. Graph-based models that assume that genes with high phenotypic similarity associate with the same disease [14] also rely on highly incomplete data that can lead to poor model performance. In addition, many models for predicting gene-disease associations construct a homogeneous graph based on a single type of data such as protein-protein interactions (PPI) [15, 16]. However, using a single type of data ignores the complexity inherent in gene-disease associations [17, 18]. For example, combining PPI with tissue-specific data is important for predicting disease genes [5].

In recent years, researchers have represented nodes as numeric vectors (embeddings) in a low-dimensional space while preserving node and graph topological similarity using neural networks [19]. These embedding vectors allow ML methods to predict disease genes in graphs [20], among other tasks. Automatic feature learning from graphs [19, 21–24] has been widely studied. Graph embeddings were successfully used in capturing the biological structures of proteins [25]; reducing data noise in graphs [26] by using tasks such as node classification, link prediction, and clustering [21]; and detecting drug-drug side-effects [27].

Node embedding models for identifying disease genes are limited by relying on gene-phenotype associations, which are highly incomplete in humans and other organisms [28]. In addition, data about gene-disease associations is often limited since these complex relationships are rare, and are usually not observed in small clinical trials, preventing ML models from learning these associations [18]. Moreover, ML models for predicting disease genes are typically approached using a binary classification of gene and disease association, by selecting a sample of (i) gene-disease edges as positive training samples, and (ii) non-associated gene and disease nodes (representing non-existing edges) as negative examples that might contain unknown disease genes. Training an ML model on those negative samples may result in poor model performance.

Another limitation of most gene-disease prediction models involves assuming that accurate performance on the test set leads to accurate predictions of novel gene-disease associations. However, predicting novel disease genes requires considering *all* potential associations between candidate genes and a disease, beyond selected gene-disease associations in the test set. The accuracy of past models when considering all candidate genes is typically supported by manually examined literature without systematic evaluation. Consequently, given a specific disease, we do not know whether the predictions of novel genes by past models are accurate enough to be validated in wet-lab experiments, even though those models perform well on a test set.

Lack of studies that consider in a single model knowledge from both local network neighbors and non-neighbors; incorporate rich knowledge from several biological domains; and address the problem of sampling negative edges for link prediction raises the need for developing new models that will enable better prediction of disease genes. We address those limitations by developing a model to improve the prediction of disease genes: we propose a Heterogeneous Integrated Graph Model for Predicting Disease Genes (HetIG-PreDiG) with gene prioritization based on gene expression. HetIG-PreDiG (pronounced “HET-ih-jee PRED-ih-jee”) detects human gene-disease associations by integrating data about gene expression in different tissues, and gene-gene, gene-disease, and gene-tissue associations into a heterogeneous graph. Using the node2vec algorithm [21], HetIG-PreDiG accounts for graph structure by learning low-dimensional representation embeddings of nodes. To extend beyond graph structure, a Gene-Disease Prioritization Score (GDPS) is developed. This GDPS reflects the association degree of a gene with a disease based on co-expression similarity across multiple tissues. Node embeddings and the GDPS are input as features to a classifier that predicts gene-disease edges. To train the classifier, we randomly select gene-disease edges as positive samples, and non-associating gene and disease nodes with lower GDPS as negative training samples, thus lowering the risk of including biologically existent yet to-date unreported disease genes. The results show that a model that considers both network structure and GDPS outperforms other baseline models. Finally, we provide a method to systematically evaluate the developed model’s success in predicting novel disease genes by classifying a disease based on its candidate genes’ prediction probabilities into three success level groups.

We make five novel contributions to improve the identification of human gene-disease associations: 1) developing our Gene-Disease Prioritization Score (GDPS) based on data of gene co-expression similarity. Whereas most models for predicting disease genes use gene expression to identify genes having expression most strongly associated with a disease, our model uses it together with node embeddings for learning the degree of association between any gene and a disease; 2) considering network structure using graph representation learning, and extending beyond network structure by accounting for biological similarity between unconnected gene and disease nodes using GDPS; 3) offering a solution to the problem of randomly selecting as negative training samples non-associated gene and disease nodes (reflecting non-existing edges) that might actually represent yet-unknown biological associations, by our method of favoring non-associating gene and disease pairs having lower GDPS values; 4) capturing rich biological knowledge in a single heterogeneous graph-based model, the utility of which for predicting gene-disease associations is validated by producing better results compared with baseline models, and via literature analysis; and 5) providing a new method to systematically evaluate the developed model’s success level in predicting novel genes for a given disease, as the accuracy of past models that consider all candidate genes is typically supported via literature analysis, but these results are often not systematically evaluated as in the current work.

### 1.1 Organization

Section 2 provides a detailed overview of existing methods for predicting disease genes, and the shortcomings of those methods. Section 3 describes the HetIG-PreDiG model for predicting human disease genes, and the datasets used for learning and predicting gene-disease associations. Section 4 details the analyses performed with the developed model. Section 5 discusses the results of comparing the proposed model with baseline models, empirically evaluating the proposed model, and demonstrating its usefulness via supporting literature analysis. Section 6 discusses the strengths and limitations of this work, and interprets aspects of our results. Finally, Section 7 summarizes the contributions of this study and describes potential additional applications of our model.

## 2 Related work

### 2.1 Biomedical data

Biomedical data is often high-dimensional, incomplete, and biased due to e.g., physical measurement limitations and technological constraints [14, 26, 29]. To better understand complex biomedical phenomena such as diseases, an effective model must incorporate diverse biomedical datasets from different domains [27]. ARCHS4 [30] is a web resource that provides co-expression similarity matrices of human and mouse genes, based on RNA-seq data processed from Gene Expression Omnibus (GEO) [31]. This data can be used to detect biological functions such as gene-disease associations [30]. For example, Lachmann et al. [30] found that genes with highly correlated expression tend to share biological functions. Moreover, the authors were able to predict gene function using the extensive expression data available from ARCHS4. Other biological data sources with relevance for understanding human diseases and their treatment include the DisGeNET database [32], which provides data on Mendelian, complex, and environmental human diseases. Additionally, the Human Protein Atlas [33] serves as a map of the human proteome, providing tissue-specific gene expression data that can be used to elucidate the mechanisms of disease [34].

Building models using incomplete data can cause them to perform poorly given new data. Thus, predicting gene-disease associations for a genetic disease requires analyzing genes that are associated with the disease and genes that are likely to associate with the disease, as well as their interactions in diverse biological functions [35].

### 2.2 Predicting gene-disease associations

The genome-wide association study (GWAS) [36] is a widely used approach that analyzes single nucleotide polymorphisms (genetic variations) among humans for predicting new disease genes. However, predicting gene-disease associations via laboratory experiments and statistical analyses is time consuming, and often results in a large number of candidate genes with multiple false positives [37]. Moreover, GWAS mainly focuses on gene-phenotype associations, excluding the functions of biological molecules that act via complex pathways [37]. To address this gap, researchers have developed computational approaches such as networks for predicting gene-disease associations. Analyzing biological data using graphs can identify complex interactions among entities (e.g., genes and diseases), and it facilitates the detection of variations (e.g., genetic mutations) via structural changes in the graph [3–5].

Different types of graphs have been exploited for predicting disease genes [17], including homogeneous [3, 16, 38], heterogeneous [39], and multiplex graphs [40]. A homogeneous graph includes nodes and edges each of a single type, a heterogeneous graph has different types of nodes and edges, and a multiplex graph is a collection of graphs with the same set of nodes and different types of edges.

Two network-based approaches are commonly used for predicting disease genes. *Node classification* learns features of known disease genes to predict the disease labels of genes of novel disease associations. *Link prediction* learns known gene-disease associations to predict novel gene-disease links. These two network-based approaches for predicting disease genes can be implemented via three categories: 1) network diffusion, 2) supervised ML methods in which features for diseases and genes are first extracted and then input to ML models such as Support Vector Machines (SVMs) for predicting gene-disease associations, and 3) graph representation learning.

The next subsections describe each of these methods.

### 2.3 Network diffusion methods

Most network-based methods assume that genes associated with biologically similar diseases have similar network structures [6, 41]. Some network methods for predicting disease genes consider only the local neighborhood of a node [3, 10, 38, 42], thus missing biological information at greater distances on the network. This limitation is partially resolved by network diffusion models that start from known disease genes and diffuse to other nodes via walks over the edges of the biological network. For example, the Random Walk with Restart (RWR) algorithm [43] performs a random walk on a graph with a restart probability *r* to return to any seed node at each iteration. It explores the neighborhood of seed nodes to study their functions, under the assumption that nodes related to similar functions are closer in the network. Adopting RWR, PRINCE [44] expands RWR to a weighted PPI network, and VAVIEN [7] prioritizes candidate disease genes based on the topological similarity of proteins that is calculated using RWR to perform random walks on a PPI network. RWR was widely used in PPI networks to detect novel disease genes. For example, ORIENT [45] uses RWR to detect novel disease genes in a weighted PPI network such that genes closer to known disease genes receive a higher prioritization score. The DP-LCC model [46] also detects novel disease genes using RWR on a PPI network and a phenotype similarity network.

Heterogeneous networks of gene-disease associations, disease-disease similarities, and protein–protein interactions have also been employed for predicting disease genes. For example [39], detects disease genes in heterogeneous networks using diffusion and node classification. Other examples include the RWRH model [47] that extends RWR on a heterogeneous phenotype-gene network. RWPCN [48] predicts disease genes on a heterogeneous network of phenotypes, genes, and proteins. CIPHER [49] predicts unknown disease genes in a heterogeneous network using phenotype similarity and gene proximity. The CATAPULT model [50] predicts gene-phenotype associations by vectors generated using walks on the heterogeneous network of gene-gene and gene-phenotype associations. BiRW [51] performs RWR on a heterogeneous network of phenotypes and genes. RWRMH [40] conducts RWR on a multiplex heterogeneous network of PPIs and disease associations based on phenotype similarities. Zeng et al. [52] proposed a latent factor method with heterogeneous similarity regularization to predict unknown gene-disease associations.

While network diffusion models utilize network structure to capture biological information beyond local neighborhood, they miss global information beyond network structure, involving unconnected nodes. Networks represent existing datasets, reflecting only known (often incomplete, noisy, and biased) data collected so far [4]. Hence, the information of, e.g., two unconnected proteins in a PPI network that might share the same biological pathway [15] is ignored in diffusion models. On the other hand, gene expression data can be used to calculate similarities among nodes representing biological entities [30] and is not limited by node connectivity. However, it ignores the structure of the network.

Some studies utilized gene expression data to detect disease genes, such as Hu and Agarwal [53], who begin by identifying the top genes having expression most strongly associated with each disease. Then, they perform enrichment analyses to find significant overlaps between these top genes and diseases. Another example is the DiseaseConnect web server [54] that utilizes gene expression profiles, gene-disease associations, and GWAS data to detect novel gene-disease associations.

Recently, biological network-based models have represented nodes using feature vectors of structural network properties, such as average distance to disease genes, and structural similarity with disease genes [17, 55]. These vectors are used for training supervised ML models such as Logistic Regression (LR) and SVM to classify genes as associated with a disease or not [50, 56].

### 2.4 Supervised machine learning methods

Current ML methods that are applied to biological graphs typically represent genes and proteins using feature vectors of structural network properties (e.g., degree) [55]. ML models that use PPI networks to predict unknown disease genes include, e.g., the gene ranking model described in [57] that prioritizes candidate genes using network analysis of their differential expression. Relying on network structure, it assumes that candidate disease genes are neighbors of highly differentially expressed genes. More recently, BRIDGE [58] was developed to prioritize disease genes by applying Lasso Regression to a variety of biological resources, including PPI, protein sequence, gene expression, pathway, and gene ontology data. Similarly, the IMRF [59] algorithm also utilizes diverse biological data such as PPI networks to rank disease genes by improving the Markov Random Field method. Focusing on gene-disease association data, the Know-GENE algorithm [60] prioritizes candidate genes associated with a disease by calculating gene-gene similarity using gene co-occurrence. The authors recommend [60] considering gene expression data in future work to detect genes without known disease associations, as done in the current study. Representing diverse biological data as heterogeneous graphs, Metagraph+ [61] predicts disease genes by analyzing a heterogeneous graph of PPI and gene keywords. Using gene ontology similarities, the dgMDL algorithm [62] predicts gene-disease associations in a heterogeneous PPI and gene graph. The Disjunctive Graph Integration model [63] predicts novel disease genes by applying SVM to features of a heterogeneous graph of gene co-expression, pathways, functional links, phenotype similarity, and PPI.

Other data sources than PPI networks, such as disease-phenotype associations, gene ontology annotations, and tissue-specific networks, have been utilized to predict disease genes [10, 17, 42]. The use of tissue-specific gene expression data is critical, because diseases are typically associated with a specific tissue [64]. For example, NetWAS [65] analyzes a network of genes and tissue expression data to identify disease associations.

Some of the reviewed ML models in the current section require the handcrafted generation of graph features (e.g., distance between a gene and a disease) for training a model to classify genes as likely to be associated with a disease or not. Handcrafted feature generation is time consuming and requires domain knowledge. In contrast, graph representation learning methods [19, 21, 22] automatically learn graph features, as discussed next.

### 2.5 Graph representation learning

Automatic feature learning from graphs [17, 19, 21–24] has been widely studied using methods such as matrix factorization and graph embeddings.

**Matrix factorization methods** are used for predicting previously unknown gene-disease edges. For example, the PCFM algorithm [24] uncovers hidden factors for genes and diseases from a gene-disease association matrix using a probability-based collaborative filtering model to predict disease genes. Manifold learning [66] utilizes a gene-disease association matrix to learn latent factors of genes and diseases, following the assumption that disease genes are closely located on the graph. Medusa [67] analyzes 16 heterogeneous graphs as matrices to establish connections between non-neighboring nodes in each graph. GeneHound [68] first integrates data including literature-based phenotype and gene information. Then, it performs Bayesian matrix factorization to uncover latent factors for genes and diseases to predict new gene-disease associations.

**Graph embedding methods** represent nodes as numerical vectors in a low-dimensional space while preserving node and graph topological similarity using neural networks [19]. The goal of graph embedding methods is to capture the topological information of nodes and edges. Graph embeddings were successfully used in capturing the biological structures of proteins [25] and reducing data noise in graphs [26] by using tasks such as node classification, link prediction, and clustering [21].

Examples of node embedding algorithms include the SkipGram algorithm [69] that constructs associations between a node and its neighbors via random walks. DeepWalk [23] expands SkipGram to perform random walks on a graph by treating nodes as words. It was used to learn node embeddings in biological graphs for tasks such as predicting drug-target associations [70] and protein function [71]. SmuDGE [28] expands SkipGram to predict novel disease genes by combining disease–phenotype and gene–phenotype associations to generate a corpus for SkipGram-based representation learning. Then, it predicts gene–disease associations using a neural network. Building upon SkipGram, HeteWalk [20] constructs a weighted heterogeneous network by joining six public data sources including PPI, miRNA similarity network, and disease phenotype similarity network, and then performs SkipGram-based network embedding. The HIN2Vec algorithm [72] generates node embeddings for heterogeneous networks based on random walks using a three-layer neural network model, but it samples only short paths, making it inefficient for large graphs [73].

The node2vec algorithm [21] finds an embedding function such that the conditional probability of observing the neighbors of a node is maximized. It extends DeepWalk, but employs more sophisticated random walks using four parameters to select the next visited nodes: 1) number of random walks from each node, 2) walk length, 3) *P*—the probability to return to a previously visited node, and 4) *Q*—the probability to explore undiscovered nodes. The node2vec algorithm is widely used for generating node embeddings, and it presents superior performance in node classification tasks on biological networks [74]. Leveraging node2vec, several biological studies combine node2vec embeddings with other features. For example, the N2VKO algorithm [75] integrates node2vec embeddings extracted from a PPI network with biological annotations for gene-disease association prediction.

In network-based models, the structure of the network must accurately represent biological knowledge such as gene-disease associations; otherwise, feature learning will be harmed. ML models for predicting unknown disease genes might be biased because of missing gene-disease edges in the graph, due to, e.g., data that has not yet been collected (unknown). Missing edges also cause those models to ignore global information from unconnected nodes since most network-based gene-disease prediction models are limited to local node-to-node propagation. In addition, to train an ML classifier for the task of link prediction, positive (existing gene-disease edges) and negative (non-existing gene-disease edges) examples are needed. Whereas sampling positive edges from a graph is straightforward, sampling negative edges involves sampling a pair of an unconnected gene and disease. Such pairs might be biologically associated but not yet known, thus falsely used as a negative example, leading to poor model performance.

Lack of studies that consider in a single model knowledge from both local network neighbors and non-neighbors; incorporate rich knowledge from several biological domains; and address the problem of sampling negative edges for link prediction raises the need for developing new models that will enable better prediction of disease genes. We address those limitations by developing the Heterogeneous Integrated Graph Model for Predicting Disease Genes in humans (HetIG-PreDiG) model to improve the prediction of disease genes, as detailed next.

## 3 Materials and Methods

### 3.1 HetIG-PreDiG model to predict gene-disease associations

The following five steps describe the construction of the developed Heterogeneous Integrated Graph Model for Predicting Disease Genes in humans (HetIG-PreDiG) model for predicting gene-disease associations.

Step 1—**Construct a heterogeneous graph** using two types of data: (i) gene-disease associations, and (ii) gene-tissue interactions.

Using the first data type, gene-disease associations are represented as a graph, denoted by *G*_*gd*_ = (*D*_*gd*_, *V*_*gd*_, *E*_*gd*_). Nodes represent the sets of diseases *D*_*gd*_ and genes *V*_*gd*_. Edges *E*_*gd*_ represent gene-disease associations.

Using the second data type, gene-tissue interactions are transformed into a gene-gene graph *G*_*gg*_ = (*V*_*gg*_, *E*_*gg*_). Nodes *V*_*gg*_ represent genes that are connected by an edge *e*_*ij*_ ∈ *E*_*gg*_ if genes *v*_*i*_, *v*_*j*_ ∈ *V*_*gg*_ were reported in the same tissue.

Finally, both graphs are integrated into an undirected gene-disease heterogeneous graph *G*(*D*, *V*, *E*) = *G*_*gd*_ ∪ *G*_*gg*_. In *G*, nodes represent the sets of diseases *D* and genes *V*. Edges *E* represent gene-disease associations and gene-gene associations. To integrate both graphs into a heterogeneous graph, gene-gene edges *e*_*ij*_ ∈ *E*_*gg*_ were excluded if both gene *v*_*i*_ and gene *v*_*j*_ are not in *G*_*gd*_. More formally, we define: *G*(*D*, *V*, *E*) = *G*_*gd*_ ∪ *G*_*gg*_ = {(*D*_*gd*_, *V*_*gd*_ ∪ *V*_*gg*_, *E*_*gd*_ ∪ *E*_*gg*_)∣∀*e*_*ij*_ = (*v*_*i*_, *v*_*j*_) ∈ *E*_*gg*_, ∃*v*_*i*_, *v*_*j*_ ∈ *V*_*gd*_}. This allows us to include additional knowledge where: (i) at least one gene in *V*_*gg*_ is in *V*_*gd*_ and the second gene of the edge is not in *V*_*gd*_, or (ii) both genes in *V*_*gg*_ are in *V*_*gd*_.

Step 2—**Generate a labeled set**. We aim to predict missing links between unconnected gene and disease nodes. Given a network with missing links (due to yet-undiscovered knowledge), we aim to predict these missing links.

Data imbalance is a known challenge when designing machine learning models, as in the case of predicting disease genes. The abundant (majority) class contains more data than the minority class. In a gene-disease network, the number of non-associated gene and disease nodes (expressed as missing edges in the majority class) far exceeds that of disease-associated genes in the minority class. The imbalanced data presents a challenge for identifying gene-disease associations. Most traditional machine learning methods are usually biased towards the majority class, and hence lead to loss of predictive performance for the minority class. Sampling methods for dealing with imbalanced datasets are frequently used [76, 77]. We applied a sampling method of positive and negative examples that is similar to other studies that used the node2vec algorithm for representing biological entities in a graph [21, 78]. The described sampling method addresses the imbalanced learning problem by sampling an equal number of negative samples and positive samples for training [78], thus ensuring that the model is not biased toward any class.

We generate the labeled dataset of edges by following three sub-steps. First, obtain positive examples by randomly selecting 20% of gene-disease edges for each disease node in *G*, and removing them from *G*, thus generating *G*′ = (*D*′, *V*′, *E*′). This sub-step results in *N* positive examples of gene-disease edges. Second, obtain negative examples by randomly sampling an equal number (*N*) of unconnected node pairs composed of *N*/2 gene pairs, and *N*/2 gene and disease pairs. For each disease, we select non-associated genes with the lowest GDPS score (described in detail in Step 4). This process is iteratively repeated until the desired number (*N*/2) of non-existing gene-disease associations is achieved. Finally, the labeled dataset is split into Train (70%) and Test (30%) sets.

Step 3—**Learn node embedding vectors**. Previous studies (e.g., [21, 78]) found that deep learning techniques that use embedding vectors obtain better representation of biomedical entities (such as genes and diseases), and thus, improve prediction performance. We map nodes in *G*′ to a low-dimensional feature space using node2vec. The vectors of each pair of nodes *u*, *v* in the Train and Test sets are aggregated into a single vector (*u* + *v*). We chose the node2vec algorithm since it was reported to obtain richer topological representation of the network than traditional methods [78].

**Fig 1:**
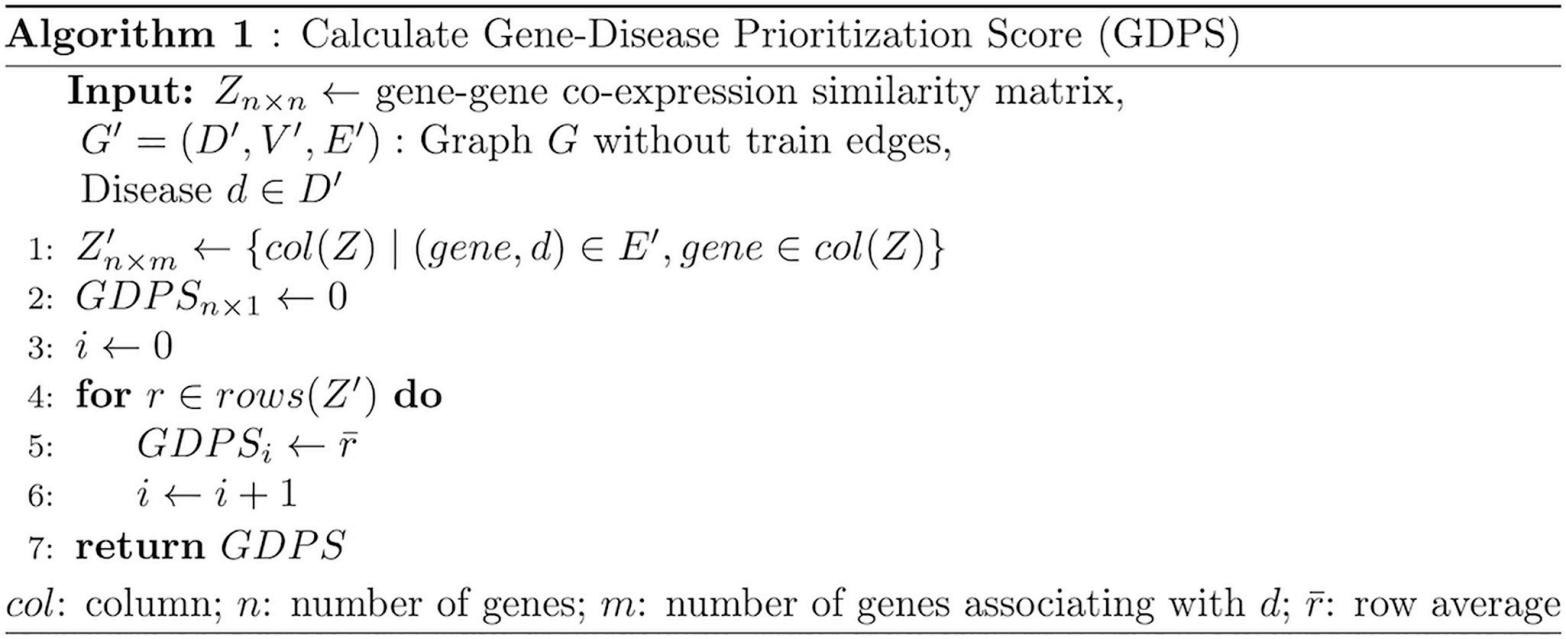
The developed algorithm to calculate Gene-Disease Prioritization Score (GDPS).

**Fig 2:**
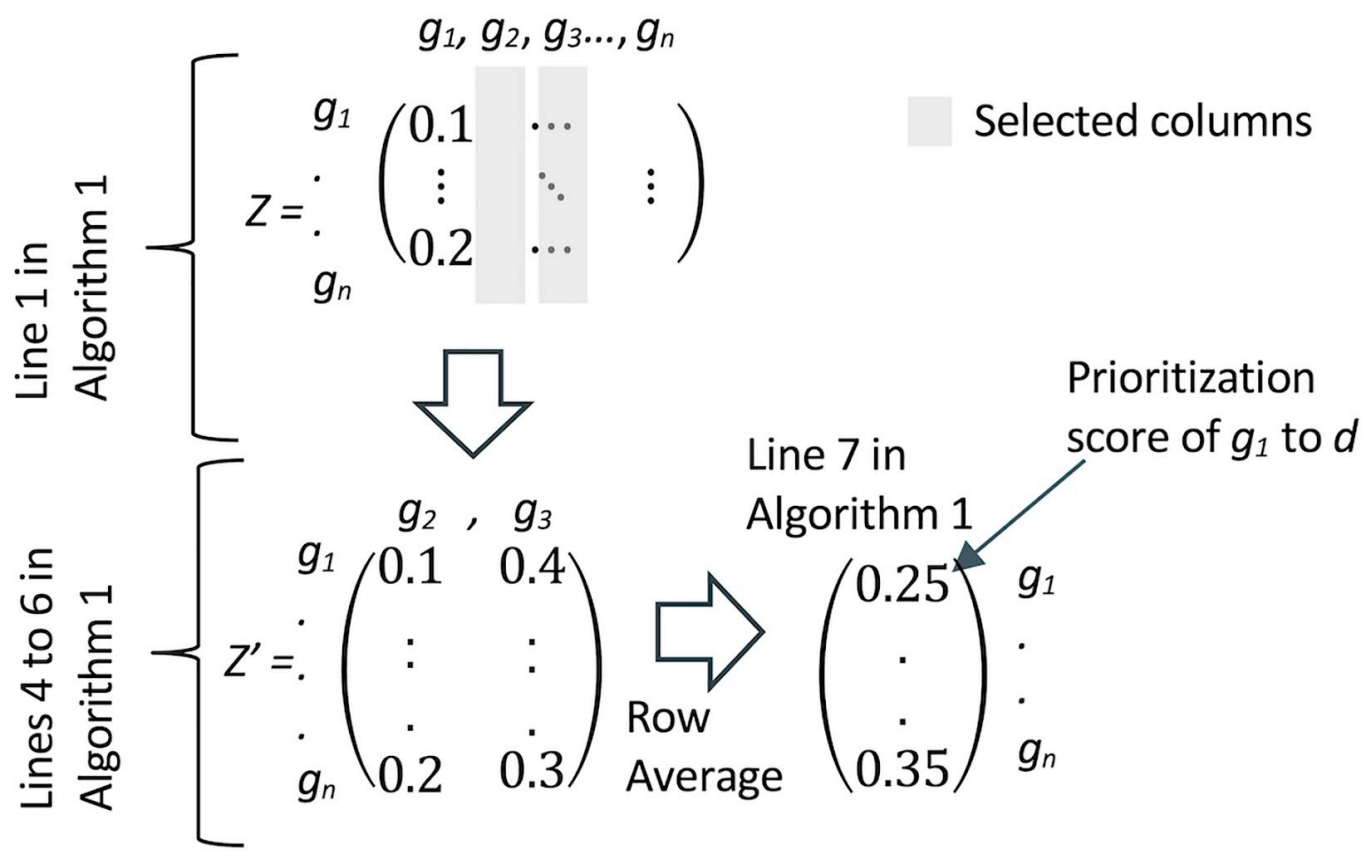
An illustrated example of Algorithm 1 (Fig 1). *Z* is a gene-gene co-expression similarity matrix. Line 1 in Algorithm 1: given disease *d* that is known to associate with genes *g*_2_ and *g*_3_, columns 2 and 3 are selected in *Z* to create matrix *Z*′. Lines 4 to 6 in Algorithm 1: each row in *Z*′ is averaged into a Gene-Disease Prioritization Score. The prioritization score of *g*_1_ for *d* is (0.1+0.4)/2 = 0.25, reflecting the average similarity of a non-associated gene *g*_1_ to genes associated with *d*.

Step 4—**Compute a Gene-Disease Prioritization Score (GDPS)** using a third type of data, gene-gene co-expression similarity. Compute GDPS using the developed Algorithm 1 (Fig 1) for each gene and disease pair, following [30]. GDPS uses a gene-gene co-expression similarity matrix to compute the average similarity of a gene to known disease genes as expressed by the structure of *G*′. The higher the GDPS value, the more likely a gene has similar functions to the disease genes, and the more likely that gene is to associate with the disease. We set GDPS for gene-gene edges to 0. When calculating the GDPS score, we use the graph *G*′, which is the original graph *G* with test edges removed. This means that only gene-disease associations that are observed in *G*′ are used to calculate GDPS. Fig 2 presents an example of Algorithm 1 (Fig 1).

Step 5—**Train ML classifier**. Concatenate the aggregated vector *u* + *v* (Step 3) with the Gene-Disease Prioritization Score (Step 4) into a single feature vector for training and testing an ML model to classify the pairs of nodes {*u*, *v*} in the Train and Test sets into one of two groups: a link will/not form. Concatenating the GDPS score to the embedding vector allows the model to learn non-linear relationships between a gene and a disease that take gene expression information into account, which is innovative [78]. To evaluate the contribution of GDPS, we compare the performance of the developed HetIG-PreDiG model with and without GDPS using 10-fold cross-validation. The best model is trained using all training examples, and then its performance is evaluated on the Test set.

### 3.2 Datasets used in model

Here we describe the datasets used for predicting gene-disease associations (Table 1).

**Table 1:** Source datasets.

Dataset	DisGeNET	Human Protein Atlas	ARCHS4
Genes	21,671	15,308	35,238
Diseases	30,170	–	–
Tissues	–	63	–
Interactions	1,134,942	653,706	(35,238)^2^

**Dataset 1 (DS1)** consists of DisGeNET V7.0 [32]. DS1 contains one of the largest publicly available collections of genes associated with human diseases curated from expert repositories, GWAS catalogs, and scientific literature. DS1 contains data including DiseaseSemanticType—the semantic type of the disease (e.g., ‘Anatomical Abnormality’, ‘Pathologic Function’, or ‘Disease or Syndrome’); Gene symbol; Disease id; Disease name; DiseaseType (‘disease’, ‘phenotype’, or ‘group’); Disease Specificity Index (DSI)—ranges from 0.25 to 1 and reflects if a gene is associated with few diseases; a gene that associates with multiple diseases has a lower DSI. Disease Pleiotropy Index (DPI) ranges from 0 to 1 and reflects if multiple diseases that associate with a gene are similar in terms of belonging to the same Medical Subject Headings (MeSH) disease class. A gene that associates with diseases of different MeSH classes has a high DPI index; and YearInitial ∈ [1940, 2020]—the year that the gene-disease association was first reported.

**Dataset 2 (DS2)** consists of the Human Protein Atlas version 20.1 and Ensembl version 92.38 with information on gene-tissue interactions. DS2 is a representative tissue-specific gene expression resource with a large and comprehensive distribution of protein-coding genes in human tissues and cells [33]. DS2 contains expression profiles for proteins in human tissues with Ensembl gene id, Tissue name, Expression level (‘High’, ‘Medium’, ‘Low’, and ‘Not Detected’), and the gene Reliability (‘Approved’, ‘Enhanced’, ‘Supported’, and ‘Uncertain’) of the expression value.

**Dataset 3 (DS3)**, the ARCHS4 database [30], covers the majority of published RNA-seq data. It contains gene counts for humans and mice from the Gene Expression Omnibus (GEO) and Sequence Read Archive (SRA) platforms. In this study, only human gene expression data is considered. Specifically, this study uses the available pairwise Pearson correlation data of human genes across expression samples to create a gene-gene co-expression similarity matrix.

## 4 Analysis

This section begins by describing the implementation of the developed HetIG-PreDiG model, following the five Method steps in Section 3.1.

In Step 1, a gene-disease graph *G*_*gd*_ based on DS1 is constructed. For DiseaseType, only ‘disease’ was selected, and for DiseaseSemanticType, only ‘Disease or Syndrome’ was selected. We removed diseases with fewer than two genes, resulting in 5,417 diseases, 13,011 genes, and 179,860 gene-disease associations.

Next, we constructed a gene-gene graph based on gene-tissue data (DS2). To consider highly validated information, only gene-tissue associations that have an ‘Approved’ Reliability with ‘High’ Expression level, and genes associating with a tissue in more than a single cell type were included. This resulted in 25 tissues, 1,661 genes, and 5,191 gene-tissue interactions. The distribution of the number of genes per tissue presents an exponential decay shape with an average of 206.64 genes per tissue, a median of 195, a maximum of 463 genes associated with the *tonsil* tissue, and a minimum of 2 genes associated with *retina*. Next, we converted the selected data into a gene-gene graph *G*_*gg*_ such that genes found in the same tissue are connected by an edge. Finally, both graphs (*G*_*gd*_ and *G*_*gg*_) are combined into a heterogeneous graph *G* with 5,417 diseases, 13,637 genes, 179,860 gene-disease associations, and 444,094 gene-gene associations.

In Step 2, given a graph *G*, we labeled a set of positive and negative examples as described in Section 3.1. This step results in the creation of graph *G*′ by deleting positive examples (edges) from *G*. Next, the examples are shuffled, and split into Train (70%) and Test (30%) sets.

In Step 3, node embeddings are learned by applying node2vec to *G*′ using the following parameters: embedding dimensions = 64, walk length = 5, number of random walks = 10, p = 1, and q = 1. For p = 1, the algorithm is less likely to revisit a node, resulting in moderate exploration and avoiding 2-hop redundancy. Setting q = 1, the algorithm is not biased towards visiting closer or farther nodes to the current node.

In Step 4, GDPS is computed using DS3 for each pair of gene and disease in the Train and Test sets.

To summarize, DS1 and DS2 were used to create nodes and edges in the analyzed network. DS3 was used to create the GDPS score. Specifically, we used gene expression level in DS2 only for selecting data for further analysis. DS3 data of gene counts (expression) is used for generating GDPS and is not used for graph structure.

Finally, in Step 5, we trained a logistic regression classifier using the Train set to classify pairs of nodes into one of two classes: 1) link formation (i.e., a gene is associated with the disease), or 2) no link formation.

### 4.1 Comparison with baseline models

To evaluate the performance of the developed HetIG-PreDiG model (Section 3.1), the Train and Test sets were used to train and evaluate the following algorithms for predicting gene-disease associations as baseline models: RWRH, N2VKO, and HIN2Vec. These models were selected since they are frequently used and were reported to produce good performance on biological datasets [21, 47, 75]. The evaluated models were assessed using the implementations described by their authors, with their suggested parameters. In addition, we performed an ablation study by comparing the performance of HetIG-PreDiG with and without GDPS using 10-fold cross-validation.

### 4.2 Model evaluation: Predicting novel disease genes

Instead of predicting specific gene-disease edges in the Test set (as in Section 3.1, Step 5), we adopt a more realistic approach in which, for a given disease, all candidate (non-associating) genes are to be examined. Stated differently, given a disease and a set of candidate genes, we aim to predict gene-disease associations. We replicate a scenario at a point in time when cumulative knowledge exists and other knowledge is missing (e.g., has yet to be discovered). Cumulative knowledge is represented by *G*′ = (*D*′, *V*′, *E*′), reflecting gene-disease associations in the Train set. We define missing knowledge as gene-disease associations discovered in the year 2020 (*DS*_2020_) (the most recent gene-disease discovery year in DS3) that appear in the Test set. We predict those associations by considering *all* candidate genes in *G*′ that are not associated with a disease *d* ∈ *D*′ ∩ *DS*_2020_. More formally, we seek to predict the following gene-disease associations {(*g*, *d*)|*g* ∈ *V*′ ∧ *d* ∈ *D*′ ∩ *DS*_2020_ ∧ (*g*, *d*) ∉ *E*′}.

We assume that gene-disease associations in *DS*_2020_ are unknown during the prediction process only. When the prediction process is complete, we evaluate our model for predicting novel disease genes (candidate genes predicted to associate with *d*), using the developed Overlap measure (1):

$$\begin{array}{c} Overlap\left(f_{\%}\right)=|P_{f_{\%}}\cap I_{d}\left|/\right|I_{d}| \end{array}$$ (1)

*P*_*f*%_—The set of candidate genes predicted to associate with *d* ∈ *DS*_2020_, located in the top *f*_%_ of a ranking, based on the gene’s prediction probability.

*I*_*d*_—The set of genes associated with *d* ∈ *DS*_2020_.

The higher the Overlap score, the more successful the developed model at identifying genes that associate with a disease. When a disease *d* ∈ *DS*_2020_ has a high Overlap score, the genes predicted to associate with *d* can be further validated in wet-lab experiments. An Overlap of 1 indicates that all genes that are known to associate with *d* were identified, and an Overlap of 0 indicates that no genes associating with *d* were identified.

Increasing *f*_%_ will result in the inclusion of more genes (e.g., *f*_%_ = 1 considers all candidate genes) leading to a higher Overlap score, but also requires experimenting with more candidate genes in a wet lab. To optimize the set of candidate genes, we define the *Ratio* (2) between the number of diseases with Overlap = 1 and *f*_%_. We aim to maximize the number of diseases with Overlap = 1 and minimize *f*_%_, i.e., find the highest Ratio.

$$\begin{array}{c} Ratio=\left|\left\{d\in Diseases|Overlap\left(d\right)=1\right\}\right|/f_{\%} \end{array}$$ (2)

We construct the set *DS*_2020_ as follows: first, we select all gene-disease associations in DS1 with YearInitial = 2020; diseaseType = ‘disease’; and diseaseSemanticType = ‘Disease or Syndrome’. Second, we remove diseases with only a single gene association discovered in the year 2020. Third, we keep only gene-disease edges that are not in *G*′. Fourth, we discard disease associations with highly connected genes (which are more trivial to predict) by selecting genes in DS1 with a DSI above the average DSI of all genes, and with DPI below the average DPI of all genes.

**Fig 3:**
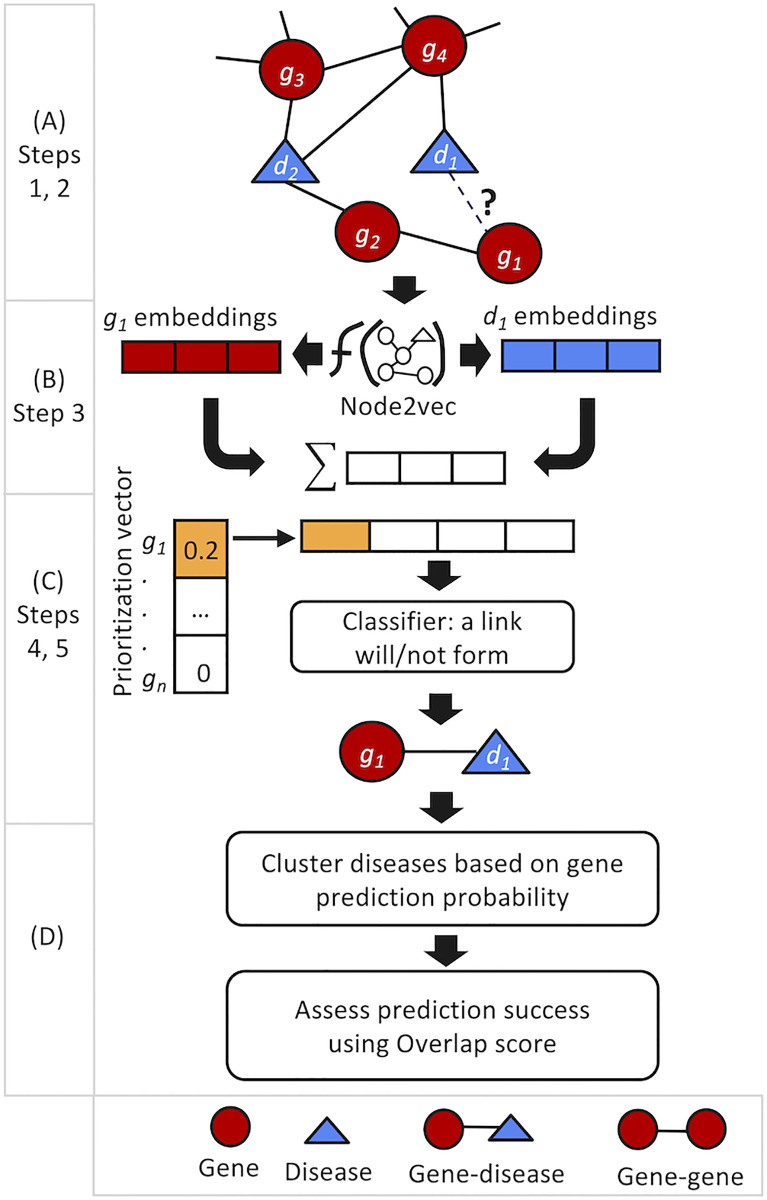
An illustration of the developed model using an example: Predict if gene *g*_1_ is associated with disease *d*_1_. (A) Steps 1,2 (Section 3.1): Heterogeneous graph (*G*′) with genes and diseases after removal of selected Train/Test gene-disease edges. (B) Step 3: Learn node embeddings. The embeddings of *d*_1_, *g*_1_ are aggregated into a single vector denoted by Σ. (C) Steps 4, 5: Compute Gene-Disease Prioritization Scores. Compute GDPS(*g*_1_, *d*_1_) in the illustrated example. Gene expression data involving gene function is integrated into the model via the GDPS prioritization score that provides expression similarity across multiple tissues. Next, the GDPS prioritization score is concatenated with the vector Σ and fed into a classifier that is trained to predict associations between pairs of genes and diseases. In the illustrated example, GDPS(*g*_1_, *d*_1_) (colored in orange) is concatenated to vector Σ. Finally, in the current example, the developed model predicts a new edge between *g*_1_ and *d*_1_. (D) Assess success in predicting novel genes: cluster a disease based on its gene prediction probabilities.

Next, we follow five steps to predict novel gene-disease associations. First, given *G*′ = (*D*′, *V*′, *E*′) and a disease *d* ∈ *D*′ ∩ *DS*_2020_, generate embedding vectors of each candidate gene *g* ∈ *V*′ not associated with *d* (i.e., (*g*, *d*) ∉ *E*′), and embeddings of *d*. Second, sum embedding vectors of each candidate gene and *d*. Third, for each candidate gene, compute its GDPS score with *d* and concatenate it to the aggregated embedding vector in the Second step. Fourth, apply HetIG-PreDiG for each candidate gene-disease pair to predict via classification whether a link will form between them (the ‘link formation’ class), or not. Fifth, keep only novel genes that are predicted to associate with *d* (i.e., classified into the ‘link formation’ class) with their probability of affiliating with that class (prediction probability is used later in Section 5.3). Fig 3 summarizes the steps of model evaluation.

Next, given a disease, we estimate the success of our ‘HetIG-PreDiG with GDPS’ model’s success in predicting gene-disease associations without the ability to calculate the Overlap score.

### 4.3 Model evaluation: Real-world scenario

In a real-world scenario, there is no test set. We can predict novel gene-disease associations similar to Section 4.2, but we cannot calculate the Overlap score (*I*_*d*_ is unknown). Hence, we cannot evaluate our predictions’ correctness. Given a single disease, we aim to estimate the prediction performance of our model in identifying novel gene-disease associations. While model performance is typically evaluated based on the prediction success of the entire test set (e.g., F1 score), a single prediction might be wrong (e.g., false positive); thus, we do not know if our single prediction is successful.

To overcome this challenge, we identify via classification, diseases that are more likely to have higher Overlap values. We learn the patterns of the prediction probabilities of genes that were predicted to associate with diseases in Section 4.2. Prediction probabilities have been found informative for biological problems such as estimating the plasma effect-site equilibration rate constant [79] and anesthetic depth [80]. Under the assumption that prediction probabilities contain meaningful information about the Overlap score, we learn their patterns by: (i) calculating the mean prediction probability of gene-disease associations for each disease *d* ∈ *DS*_2020_; (ii) analyzing the mean prediction probabilities for diseases *d* ∈ *DS*_2020_ to automatically detect the optimal number of clusters using the Ckmeans.1d.dp R library [81] that is a variant of K-means for one-dimensional data; and (iii) classifying each *d* ∈ *DS*_2020_ to clusters of different Overlap range using the Ckmeans.1d.dp R library.

To summarize, using the ‘HetIG-PreDiG with GDPS’ model, we first predict the genes associated with a disease. Then, using the developed classifier in Step *iii*, we can classify the disease based on its prediction probabilities and estimate its Overlap score based on *d*’s cluster affiliation. For high Overlap (∼ 1), experiments in a wet lab may be warranted.

We make predictions for each *d* ∈ *DS*_2020_ using all candidate genes in the dataset. Then, we determine whether the top-ranked genes predicted by the developed model are novel by conducting an automated literature search to find papers indexed by PubMed that support the predicted gene-disease associations. Using the PubMed API, we collect papers containing the disease’s name and its top-ranked predicted genes together within the title and/or abstract fields. We further validate the reported papers by manually examining the complete set of results for a subset of the diseases.

### 4.4 Comparison of gene-disease association predictions with the literature

In this analysis, the predictions of HetIG-PreDiG are evaluated by demonstrating its capability to predict novel gene-disease associations that are not present in DS1. Studies are presented that support the highest top-ranked genes predicted to associate with a disease. We focus on the diseases in *DS*_2020_. For each of the Top 10 predicted genes for each disease, we searched for supporting literature to determine whether those genes are novel using automated and manual searches.

Using an automated search, we surveyed the literature using PubMed’s API as described in Section 4.3 to find existing associations between each disease and its Top 10 predicted genes. To evaluate the developed model’s success in detecting novel disease genes, we calculated the *Success Rate* defined by the ratio between the number of genes with supporting literature evidence and the number of top-ranked genes (i.e., 10).

## 5 Results

### 5.1 Comparison with baseline models

The prediction performance of the HetIG-PreDiG model outperformed the prediction performances of the baseline models, as listed in Table 2, which reports the average and standard deviation of F1 score across the 10-fold cross-validation.

**Table 2:** Comparison of the developed model (HetIG-PreDiG) with the baseline models using 10-fold cross-validation for prediction of the top 30% of predicted disease genes. The source code of baseline models is listed. The Micro-F1 score column indicates average and standard deviation model performance of predicting gene-disease associations.

Model	Description and Source Code	F1 score
HetIG-PreDiG	The developed model with the developed GDPS score. https://github.com/bartala/disease_gene	0.95 ± 0.021
HIN2Vec	Generates node embeddings for heterogeneous networks based on random walks using a three-layer neural network model, but it samples only short paths, making it inefficient for large graphs. https://github.com/csiesheep/hin2vec	0.89 ± 0.036
HetIG-PreDiG no GDPS	The developed model with node embeddings only, without Gene-Disease Prioritization Score (GDPS).	0.88 ± 0.028
N2VKO	Integrates node2vec embeddings extracted from a PPI network with biological annotations for gene-disease association prediction. https://github.com/sezinata/N2VKO	0.82 ± 0.131
RWRH	Extends RWR on a heterogeneous phenotype-gene network. https://github.com/alberto-valdeolivas/RWR-MH	0.79 ± 0.058

**Fig 4:**
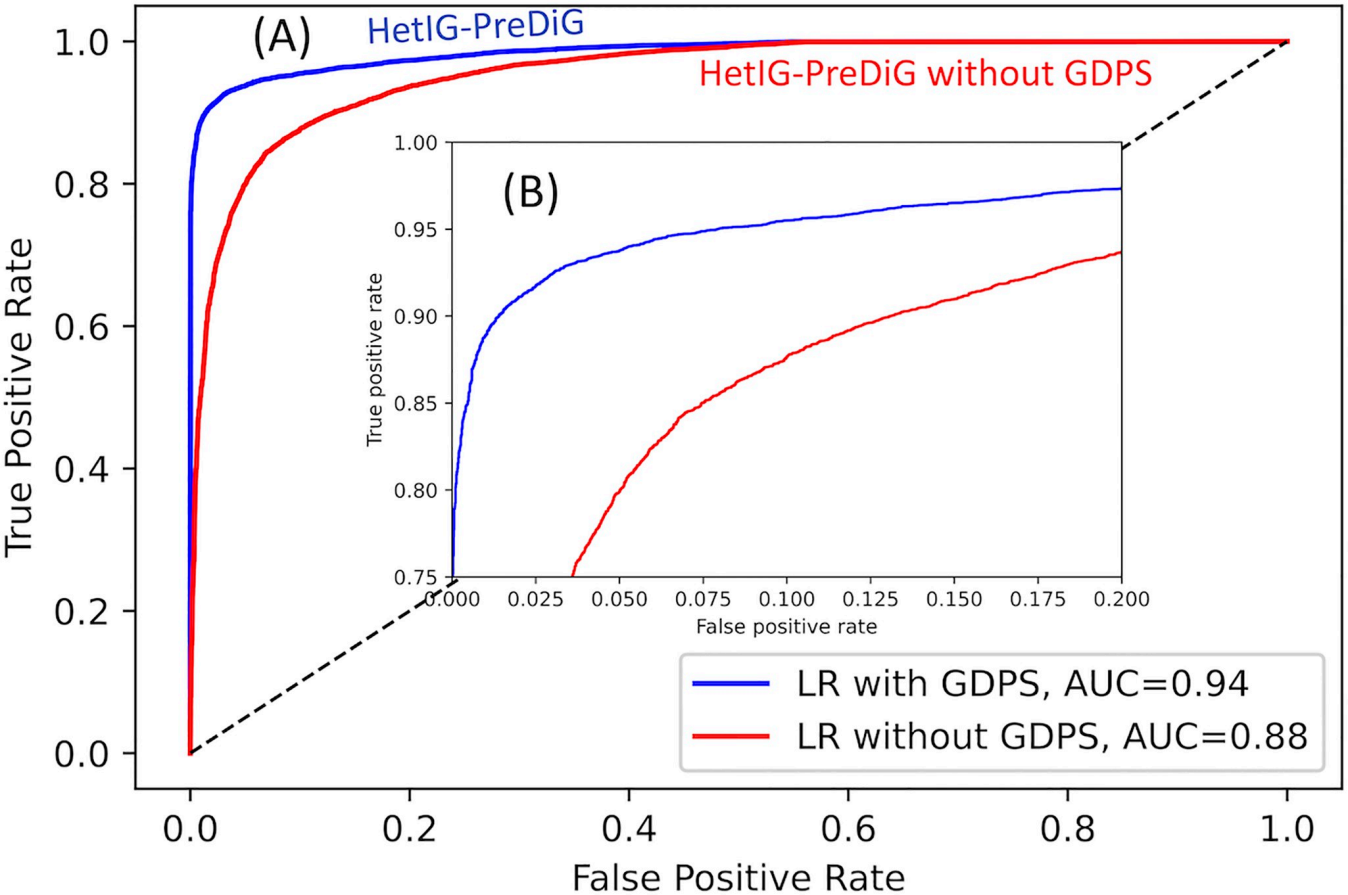
An ablation study. Demonstrating the effectiveness of using the developed Gene-Disease Prioritization Score (GDPS) in the developed HetIG-PreDiG model that incorporates a logistic regression (LR) classifier. (A) Receiver operating characteristic (ROC) curves of the developed model with (blue), and without (red) GDPS. The area under each ROC curve (AUC) is indicated. (B) A zoomed view of the top left corner of Fig 4A.

Regarding the ablation study, HetIG-PreDiG with GDPS outperformed HetIG-PreDiG without GDPS (Table 2). When evaluated on the Test set, HetIG-PreDiG with GDPS outperformed (Recall 0.93, Precision 0.97, and Micro-F1 score 0.95) the HetIG-PreDiG model without GDPS (Recall 0.87, Precision 0.88, and Micro-F1 score 0.88). Fig 4 shows model performances evaluated using the Receiver Operating Characteristic (ROC) curve, and the Area Under the ROC Curve (AUC). The addition of the GDPS score improves the prediction performance of model ‘HetIG-PreDiG with GDPS’ compared with model ‘HetIG-PreDiG without GDPS’ (Fig 4). The HetIG-PreDiG model developed in this study considers information not only from the structure of the network using node embeddings but also from biological information not reflected in the network, using gene co-expression similarity.

The next section demonstrates the capability of the developed HetIG-PreDiG model for predicting novel gene-disease associations.

### 5.2 Model evaluation: Predicting novel disease genes

**Fig 5:**
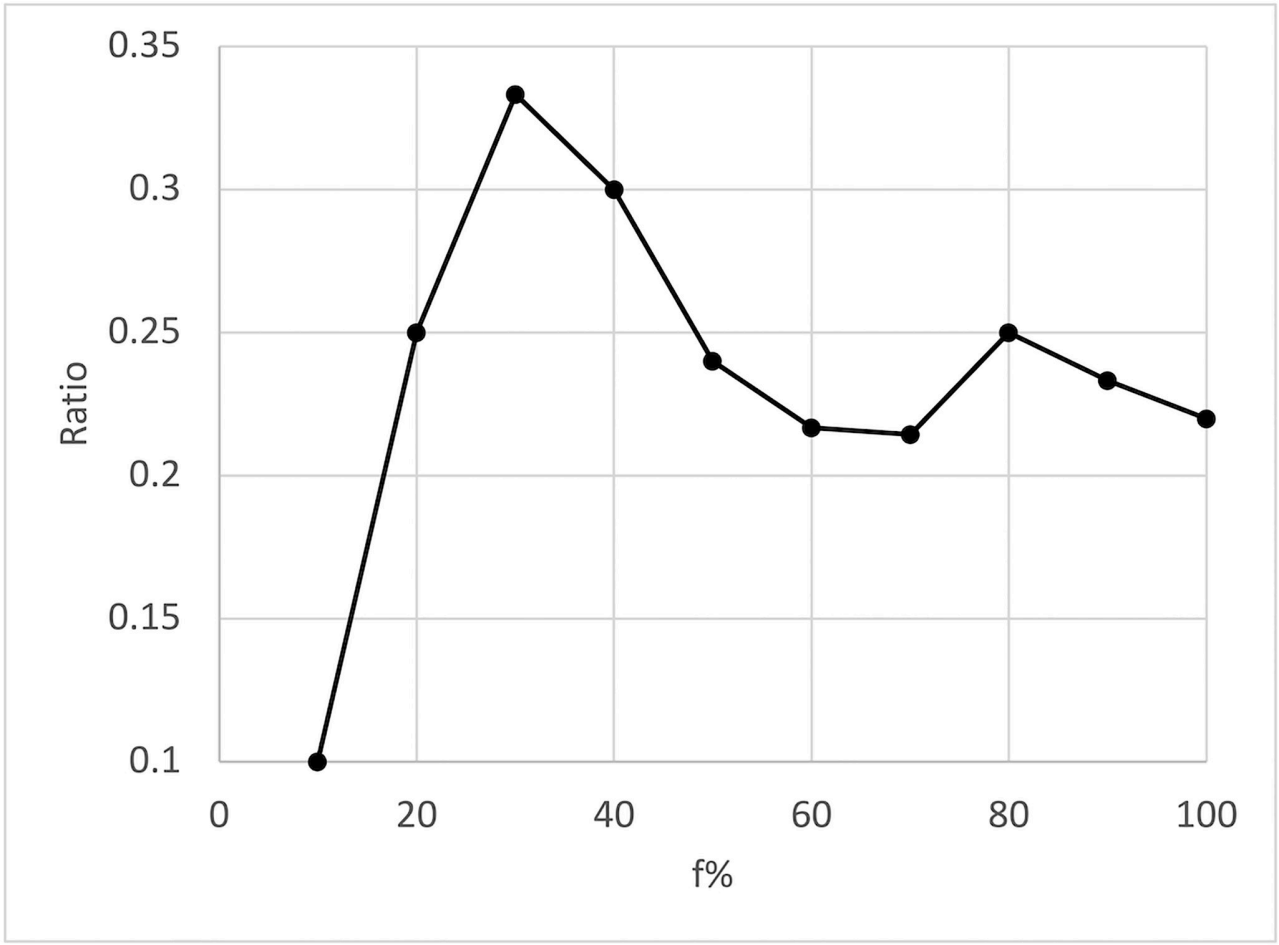
Ratio between the number of diseases with Overlap = 1 and *f*_%_. The highest ratio is achieved at *f*_%_ = 30%.

To evaluate the model’s success in predicting novel gene-disease associations, we followed the steps described in Secion 4.2. This resulted in 30 diseases in *DS*_2020_ (Table 3) associating with 11,667 genes. For each disease *d*, we computed the Overlap score for different *f*_%_ ∈ [10, 100] rankings, with steps of 10. We focus on *f*_%_ = 30% since it presented the highest Ratio in Eq. (2) as illustrated in Fig 5. The Overlap score was 1 for 10 diseases; it was in the range (0.5, 1) for 2 diseases; and it was in the range [0, 0.5] for 18 diseases, indicating the identification of all, most, and few associating genes predicted for *f*_30%_, respectively. As expected, we find that the Overlap score increases as the *f*_%_ increases. In contrast to *f*_%_ = 30%, at *f*_%_ = 100%, the model predicts an Overlap = 1 for 22 diseases, Overlap ∈(0.5, 1) for 4 diseases, and an Overlap ∈[0.5, 1] for 4 diseases.

Table 3 provides a summary of the literature evidence for the Top 10 predicted genes not associated with a disease for each of the selected 30 diseases. The Success Rate column is the ratio between the number of predicted genes with PubMed supporting evidence divided by 10. For example, for the disease Anemia, 9 of the Top 10 predicted genes for this disease had PubMed literature support, with a total of 453 PubMed entries supporting these 9 gene-disease predictions.

**Table 3:** A summary of literature evidence for the Top 10 predicted genes that are not associating with a disease for each of the selected 30 diseases. The Success Rate column is the ratio between the number of predicted genes with PubMed supporting evidence divided by 10.

Disease Name	Success Rate	Supporting PubMed Papers
Alzheimer’s Disease	1.0	91
Anemia	0.9	453
Anorexia	0.9	385
Arthritis	0.9	551
Obesity	0.9	102
Parkinson Disease	0.9	76
Colitis	0.8	992
Dermatitis	0.8	243
Diabetes Mellitus	0.8	150
Myocarditis	0.8	33
Degenerative Polyarthritis	0.8	23
Rheumatoid Arthritis	0.8	177
Sepsis	0.8	204
Crohn’s Disease	0.7	52
Hyperglycemia	0.7	117
Liver Fibrosis	0.7	90
Alopecia	0.6	134
Coronary Artery Disease	0.6	13
Diabetic Nephropathy	0.6	12
Hepatitis B	0.6	17
Hepatitis C	0.6	17
Retinitis Pigmentosa	0.6	28
Cerebral Infarction	0.5	30
Hypertrophic Cardiomyopathy	0.5	49
Idiopathic Pulmonary Fibrosis	0.5	35
Retinopathy of Prematurity	0.5	6
Anophthalmia and Pulmonary Hypoplasia	0.4	4
Chagas Disease	0.4	18
Septicemia	0.4	62
Amyloidosis	0.3	23

### 5.3 Model evaluation: Real-world scenario

Using the prediction probability results of Step 5 in Section 5.2, we learn the patterns of the prediction probabilities of candidate genes predicted to associate with diseases *d*. Following Step *i* (Section 4.3), we calculate the mean prediction probability of gene-disease associations for each *d* ∈ *D*′ ∩ *DS*_2020_. In Step *ii*, we analyze the mean prediction probabilities for diseases *d* and automatically detect the optimal number of clusters using the Ckmeans.1d.dp R library [81]. The search for the optimal number of clusters was performed in the range of 2 to 9. Three clusters were identified. Lastly, in Step *iii*, we assign each *d* to a cluster based on its mean prediction probability.

Based on the Overlap scores (calculated in Section 5.2) of diseases in each cluster, the clusters were assigned a respective range of Overlap: (a) 0 ≤ Overlap ≤ 0.5, (b) 0.5 < Overlap < 1, and (c) Overlap = 1 to capture (a) few, (b) most, and (c) all associating genes predicted in *P*_*f*%_, respectively.

**Fig 6:**
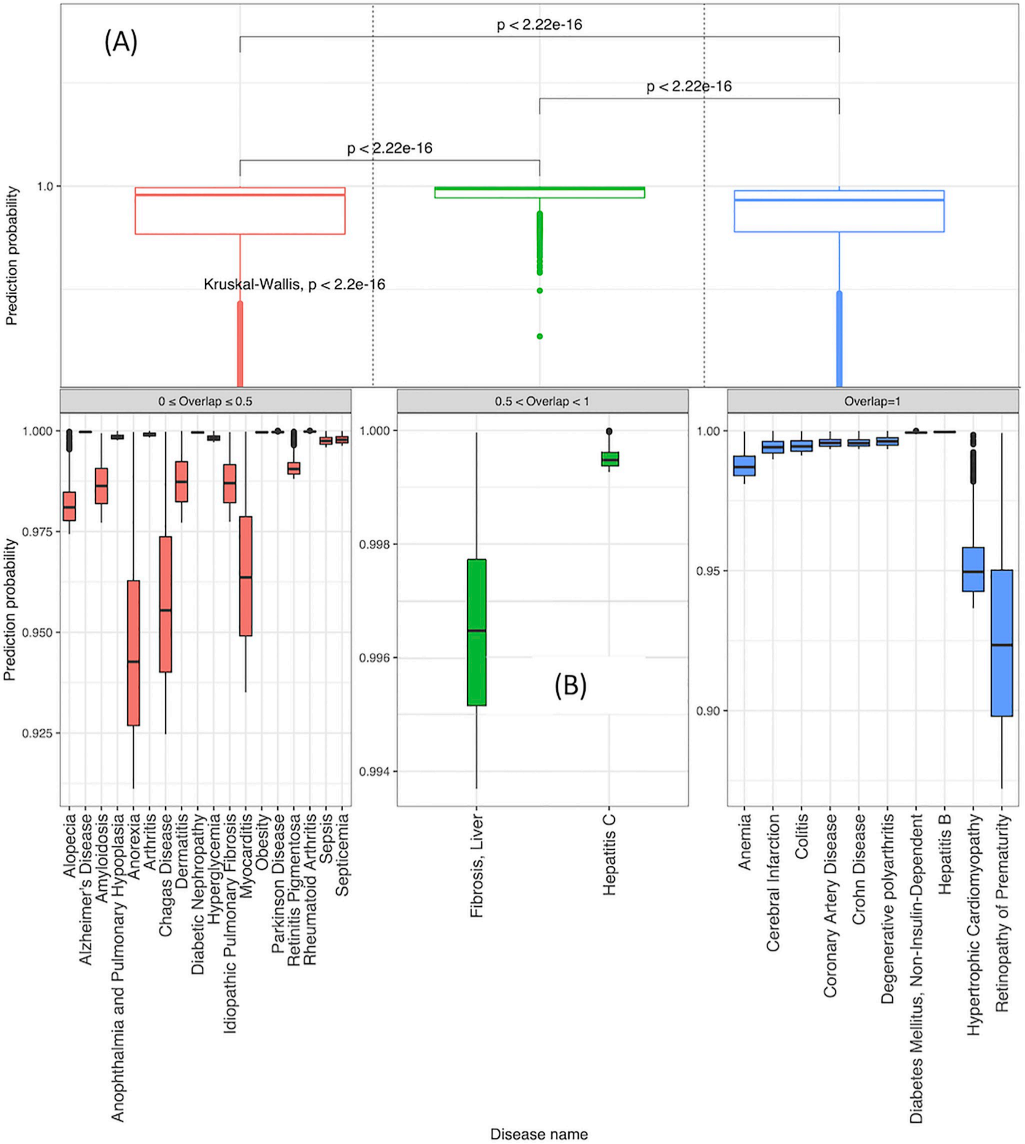
Box plots of the top *f*_%_ = 30% prediction probabilities of genes predicted to associate with a disease. (A) three levels of Overlap clusters, and (B) a breakdown showing the diseases affiliating with each cluster in (A).

Fig 6A presents the prediction probabilities distributions of an Overlap score for the top *f*_%_ = 30% in each of the three clusters. Using a Kruskal-Wallis rank (KW) test [82], we find significant differences (*P*_*value*_ < 2.2*e*^−16^) in the density for the three classes. To detect where those differences lie, we conducted three additional KW tests between each pair of classes, and again find significant differences. Fig 6B presents a breakdown of the diseases that affiliate with each cluster in Fig 6A.

To summarize, given a disease, in a real-world scenario where the Overlap score cannot be calculated, we first predict disease genes, and then assign the disease to one of the three Overlap clusters based on the mean gene prediction probabilities. While all models make mistakes, our clustering method enables us to systematically estimate the model’s success in predicting novel disease genes.

While the developed model can successfully predict gene-disease associations, other predicted genes for a given disease *d* that were not yet validated might also associate with *d*, as demonstrated next.

### 5.4 Comparison of gene-disease association predictions with the literature

Table 3 presents a summary of the 30 diseases in *DS*_2020_, their corresponding Success Rates, and the number of supporting PubMed papers reported. Using a manual search, we reviewed a subset of the 30 diseases in Table 3 for which ≤100 publications were reported.

Representative of the developed HetIG-PreDiG model’s gene-disease association prediction quality, Table 4 provides reported literature support for the Top 10 predicted genes from the developed model, for three of the diseases listed in Table 3: Hepatitis B, Chagas Disease, and Diabetic Nephropathy. We selected those three diseases since they represent a wide range of organs and disease mechanisms involved; for organs, (i) Hepatitis B involves the liver; (ii) Chagas Disease involves multiple systems including cardiovascular and digestive; and (iii) Diabetic Nephropathy involves the kidney.

**Table 4:** Literature review for Top 10 predicted genes for 3 example diseases. Genes are listed in descending ranked order. For example, for Hepatitis B, CASP1 and BAK1 are ranked 1 (best) and 10, respectively.

Disease	Gene	Supporting PubMed Studies (PubMed IDs)
Hepatitis B	CASP1	30222506, 29803661, 24599568
	F5	NA
	PLG	33594307, 31742950, 30024701, 29384847
	MPO	11236502
	JAK2	33435880, 33249195, 32098857, 31485610, 25135878, 25931880, 23483208
	DECR1	NA
	SLC27A5	NA
	APOH	33370716
	DPP4	NA
	BAK1	32574393
Chagas Disease	VEGFA	NA
	IL4	20382097
	APOE	NA
	ICAM1	NA
	STAT3	34061845, 32892338, 31214200, 24260222, 23435997,23253440, 21984337
	IL1A	NA
	CSF2	NA
	CD14	33146244, 31379862, 27902980, 27812661, 15223603
	CXCR4	33195200, 28322302, 23597573, 16637021, 14722885
	NFKB1	NA
Diabetic Nephropathy	F2	NA
	HAMP	NA
	GPT	33232923, 16874670
	CASP1	26832955
	CSF2	31277135
	CBS	33864412
	CTNND1	21752957
	CHDH	NA
	CD14	29531593, 28058051, 25314649, 22772413, 21847775, 9498652
	MMP14	NA

Hepatitis B and Diabetic Nephropathy have literature support for 6 of the Top 10 predicted genes, and Chagas Disease is supported for 4 of its Top 10 predicted genes. All PubMed studies listed in Table 4 were manually vetted to confirm their relevance to associate each disease with its top predicted genes.

The predicted genes in Table 4 without associated literature evidence are especially interesting because these represent the HetIG-PreDig model’s predicted novel gene-disease associations. We manually examined each such predicted gene-disease association, and nearly all of these predictions proved to be reasonable based on the known functions of the genes. For example, for Hepatitis B, predicted gene #2, F5; predicted gene #6, DECR1; predicted gene #7, SLC27A5, and predicted gene #9, DPP4, are all associated with liver function by the GeneCards platform [83].

Chagas Disease, caused by infection by the parasite *Trypanosoma cruzi*, involves immune response and inflammatory lesions, and can cause heart failure, arrhythmias, and dysfunction of the digestive system [84]. Predicted gene #1, VEGFA, is involved with vasculature, and this disease often involves impairment of cardiac and vascular function [84, 85]. Predicted gene #3, APOE, has an established role in cardiac function [86]. Predicted gene #4, ICAM1, has been reported to have both cardiac [87] and digestive [88] roles. Predicted gene #10, NFKB1, has reported functions in the cardiac [89, 90], vascular [91], immune [92], and digestive [93] systems.

For Diabetic Nephropathy, gene F2 was the top predicted gene; although PubMed does not report this association, the GeneCards platform [83] associates abnormality of the kidney with this gene. Similarly, GeneCards also reports an association of kidney function with other top predicted Diabetic Neuropathy genes having no PubMed support, including HAMP (predicted gene #2) and CHDH (predicted gene #8). Predicted gene #10, MMP14, is a member of the matrix metallopeptidase/metalloproteinase family that is well established to have a role in Diabetic Nephropathy.

## 6 Discussion

This study presents a model for predicting human gene-disease associations using automatic feature learning in a heterogeneous graph with Gene-Disease Prioritization Scores, based on gene co-expression data. The developed HetIG-PreDiG model outperforms baseline models, showing that a model that incorporates this study’s novel Gene-Disease Prioritization Score (GDPS) achieves better prediction performance than models without GDPS. Biological data contained within the GDPS, based on gene co-expression data, allow the model to better capture the association between a gene and a disease.

Although the performance of the HetIG-PreDiG model for predicting gene-disease associations is promising, we note the following limitations: Theoretically, it is possible that two or more genes might have identical GDPS scores, which could result in no additional knowledge being added to the model. In addition, when manually analyzing the literature, we found that, with some exceptions, the reported PubMed publications were generally relevant to support the predicted gene-disease associations. However, we identified several challenges to the automation of this analysis, including the ambiguity of some gene names (e.g., some genes with symbols identical to acronyms that refer to an unrelated concept); the occasional reporting of a *lack* of association among genes and diseases; and the gene symbol and disease name appearing within the article’s title and/or abstract, but not being associated to each other within the study. Accordingly, because most of the diseases in Table 3 that are not included in Table 4 were not manually vetted, the Supporting PubMed Papers values in Table 3 should be interpreted with the caveat that the relevant papers represent a subset of the reported counts.

The top predicted genes for each disease warrant further examination, and potential experimental vetting. Of note is that the developed model’s predicted gene-disease associations allow both positive and negative relationships; for example, a predicted gene might either cause or increase the probability of developing the disease, or alternatively might prevent or protect against that disease. Future analyses can separate positive from negative associations, and because these effects are often context-dependent, this will require careful analysis that may necessitate the collection of additional experimental data. Future work might also enhance existing gene-disease datasets by considering novel findings not covered within the datasets that were used in this study. Future work might also benefit from adding the co-expression data as another edge layer to the network.

Advancing beyond previous models intended to predict gene-disease associations, this study presents five main contributions. First, biomedical data is incorporated in the form of a Gene-Disease Prioritization Score (GDPS) based on gene co-expression similarity, allowing the model to better evaluate the degree of association between a gene and a disease. Second, network structure is considered by node embeddings using graph representation learning, and this analysis extends beyond network structure by accounting for biological similarity between unconnected nodes using GDPS. Third, in contrast to most existing studies that randomly select non-associating gene and disease nodes that have the potential to be gene-disease associations not yet reported in the literature, we select negative training samples by favoring non-existing gene-disease edges with lower GDPS. Fourth, we show that network data combined with gene co-expression similarity data can effectively predict gene-disease associations compared with baseline models, and demonstrate this via literature analysis. Fifth, we provide a method to evaluate the developed model’s success in predicting novel disease genes.

The developed HetIG-PreDiG model can be applied to similar tasks that can be represented using networks and that benefit from incorporating gene expression data. Such tasks might include the prediction of drug-disease associations and the prediction of drug-drug interactions.

## 7 Conclusion

We have presented the Heterogeneous Integrated Graph for Predicting Disease Genes (HetIG-PreDiG) model that uses gene-gene, gene-disease, and gene-tissue associations data to generate accurate gene-disease association predictions, improving upon existing baseline models. Our model addresses the limitations of previous models for disease-gene prediction. This model has potential utility for other tasks that can be represented as networks and that involve gene expression data, including predicting drug-disease associations and drug-drug interactions.
